# Non-Suicidal Self-Injury and Pathological Buying in Community Adults and Patients with Eating Disorders: Associations with Reactive and Regulative Temperament

**DOI:** 10.5334/pb.1027

**Published:** 2020-12-28

**Authors:** Leni Raemen, Koen Luyckx, Astrid Müller, Tinne Buelens, Margaux Verschueren, Laurence Claes

**Affiliations:** 1 Faculty of Psychology and Educational Sciences, KU Leuven, Leuven, BE; 2 UNIBS, University of the Free State, Bloemfontein, ZA; 3 Department of Psychosomatic Medicine and Psychotherapy, Hannover Medical School, Hannover, DE; 4 Faculty of Medicine and Health Sciences (CAPRI), University of Antwerp, Antwerp, BE

**Keywords:** non-suicidal self-injury, pathological buying, temperament, eating disorders

## Abstract

The present study investigated the (co-)occurrence of non-suicidal self-injury (NSSI) and pathological buying (PB) and their associations with reactive/regulative temperament in a sample of female patients with eating disorders (ED) and in a sample of community adults. Our samples consisted of 254 community adults (48.8% female) and 60 female patients with ED. All participants filled out self-report questionnaires to assess NSSI, PB, and reactive/regulative temperament. Prevalence rates of NSSI and PB were respectively 14.5% and 4.8% for community women, 13.1% and 1.5% for community men, and 36.7% and 10% for female patients with ED. Only for community women, NSSI was positively related to PB. NSSI was negatively related to self-regulation in community men and women. Additionally, NSSI was negatively predicted by BAS reactivity in the clinical sample and by the interaction of BAS reactivity and self-regulation in community men. PB was predicted by low self-regulation in the three groups. Additionally, PB was positively predicted by BAS reactivity and by the interaction of BAS reactivity and self-regulation in community women. These findings indicated group differences in the (co-)occurrence of NSSI and PB and in their associations with underlying temperamental characteristics between individuals with and without ED.

## Introduction

During the past decades, there has been a growing interest in the co-occurrence of different pathological behaviours. Research has demonstrated that 25-55% of patients with eating disorders (ED) engage in non-suicidal self-injury (NSSI) (Pérez et al., 2018), and up to 12% of patients with ED engage in pathological buying (PB) (Fernandez-Aranda et al., 2008). However, although patients with ED seem more vulnerable for both NSSI and PB, research about the co-occurrence of NSSI and PB in clinical or community samples is lacking. Therefore, in the present study, we examined associations between NSSI and PB in a sample of patients with ED and in a sample of community adults. Moreover, we examined possible common vulnerability factors of NSSI and PB in both samples. Temperamental characteristics are frequently considered to be important factors underlying different types of psychopathology (Bijttebier et al., 2009). Identifying common and distinct temperamental characteristics of NSSI and PB in both clinical and community samples can lead to a better understanding of these behaviours and their co-occurrence.

Pathological buying is defined as the extreme preoccupation with buying or shopping, accompanied by irresistible impulses to buy and an inability to control the buying behaviour (McElroy et al., 1994). Whereas excessive buying/shopping in the early stages is often accompanied by positive feelings (positive reinforcement) (Weinstein et al., 2016), engagement in PB gradually becomes related to the relief of negative affect (negative reinforcement) (Kyrios et al., 2013). Moreover, when PB persists, this maladaptive behaviour can interfere with daily activities and can lead to serious distress, as well as financial, social, and interpersonal difficulties (McElroy et al., 1994). The lifetime prevalence rate of PB is approximately 5% in community adults, with younger and female adults being at higher risk to engage in PB (Maraz et al., 2016). Previous studies already demonstrated that PB has a high comorbidity with psychiatric disorders, such as bulimia nervosa and binge eating disorder (Fernandez-Aranda et al., 2008), substance abuse, and borderline personality disorder (Lejoyeux & Weinstein, 2010).

NSSI refers to the intentional and direct injury of one’s own body tissue without suicidal intent, including socially unaccepted methods such as cutting, scratching or hitting oneself (Nock, 2009). Earlier studies found NSSI to be related to psychological distress and intense negative feelings (Buelens et al., 2019) and to symptoms of depression and anxiety (Wilkinson & Goodyer, 2011). In addition, individuals engaging in NSSI have a significantly higher risk for a suicide attempt at a certain point in their life (Ribeiro et al., 2016). Lifetime prevalence rates of NSSI are 5-22% for young community adults and 2-16% for community adults above age 25 (Swannell et al., 2014). Similar to PB, longitudinal research has demonstrated that young individuals are at a higher risk for NSSI (Plener et al., 2015). Also, research has indicated that NSSI is highly comorbid with clinical disorders such as EDs (Muehlenkamp et al., 2012) and borderline personality disorder (Welch et al., 2008).

Although PB and NSSI have been demonstrated to occur frequently in patients with ED, integrative research investigating these behaviours and transdiagnostic mechanisms in community and clinical samples is lacking. To improve our understanding of NSSI and PB and their underlying mechanisms, we focus on temperamental factors. Temperament is conceptualized as individual differences in (1) *reactivity* and (2) *self-regulation* (Rothbart et al., 2000). Reactivity can be defined as the excitability and responsivity of individuals’ underlying behavioural and physiological systems (Rothbart et al., 2000). Such reactivity involves two separate underlying systems; the Behavioural Inhibition System (BIS) and the Behavioural Activation System (BAS; Gray, 1991). The BIS is activated by stimuli that signal punishment or termination of reward and results in avoidance behaviour, whereas the BAS is activated by stimuli that signal reward or the termination of punishment and results in approach behaviour (Gray, 1991). For instance, individuals with a higher tendency to avoid (BIS reactivity), are more likely to engage in behaviours to escape from negative feelings, whereas individuals with a higher tendency to approach (BAS reactivity), are more likely to engage in behaviours to acquire positive feelings.

In addition to this reactive bottom-up component of temperament, the regulative top-down component has an influence on individuals’ behavioural reactions as well. Self-regulation, also referred to as effortful control or self-control, points to the neural and behavioural processes modulating the reactivity of the BIS and BAS to obtain an adaptive behavioural reaction (Derryberry & Rothbart, 1997). More specifically, self-regulation can act as a moderator of the association between reactivity and (pathological) behaviour. Previous research has demonstrated that especially individuals characterized by high reactivity (BIS/BAS) and low self-regulation, are at risk for psychopathology (Bijttebier et al., 2009). For example, the interaction of high BIS reactivity and low self-regulation, was strongly associated with NSSI engagement in a community sample and NSSI could serve an emotion regulation function for these individuals (Baetens et al., 2011).

Existing research on the relationship between reactivity and PB demonstrated that PB is positively associated to BAS reactivity in female community adults (Claes et al., 2010) and to BIS reactivity in male community adults (Mueller et al., 2011). Additionally, PB is characterized by a lack of effortful control in both female and male community adults (Mueller et al., 2011). With respect to NSSI, previous research in community adults has shown that, regardless of gender, NSSI relates positively to BIS reactivity and negatively to effortful control. The role of BAS reactivity in NSSI is still unclear, as some results point to the absence of an association between BAS and NSSI (Gandhi et al., 2016), whereas other results demonstrated that high BAS reactivity is predictive for NSSI engagement (Jenkins et al., 2013). Finally, the association between BIS/BAS reactivity and NSSI/PB has not been investigated in ED patients before. Effortful control, however, has been shown to negatively correlate with NSSI/PB in patients with ED (Claes et al., 2014; Jiménez-Murcia et al., 2013). In sum, female community adults with a tendency to approach (BAS) and male community adults with a tendency to avoid (BIS), are more likely to engage in PB, whereas for both female and male community adults avoidance behaviour (BIS) is associated with NSSI. Additionally, low effortful control might be a common vulnerability factor for both NSSI and PB in different groups. The present study is the first to investigate if the same temperamental characteristics are associated to NSSI and PB in patients with ED and community adults.

The goal of this study is to examine the co-occurrence of PB and NSSI and their associations with underlying temperamental characteristics in a sample of community adults and a sample of patients with ED (given that PB and NSSI are frequently occurring in patients with ED). First, we determine the prevalence rates of NSSI and PB. Second, we investigate the association between lifetime NSSI and PB. Third, we examine whether NSSI and PB are associated with similar or different reactive and regulative temperamental characteristics. The three research objectives are examined and compared in three groups: female community adults, male community adults, and adult female patients with ED, as earlier studies demonstrated different prevalence rates of PB and NSSI in these groups. Based on the available literature (Fernandez-Aranda et al., 2008; Pérez et al., 2018), we expect higher prevalence rates for NSSI and PB in the ED patient sample compared to the community sample and, given their increased vulnerability for pathological behaviours, we also expect a stronger association between NSSI and PB in the ED patient sample than in the community sample. With respect to temperamental characteristics, we expect PB to be associated with low levels of effortful control and high BAS reactivity for community women and with low levels of effortful control and high BIS reactivity for community men. Further, we expect NSSI to be associated with low levels of effortful control and high behavioural inhibition (BIS reactivity) for both community women and men. For the female ED patient sample, we expect, based on existing literature (Claes et al., 2011; Claes et al., 2014), that PB is associated with low levels of effortful control and high BAS reactivity, and NSSI with low levels of effortful control and high BIS reactivity. Finally, we hypothesize that the interaction of high levels of reactivity (both BIS and BAS) and low levels of effortful control predicts the engagement in NSSI and PB in the different groups.

## Methods

### Participants and procedure

The community sample (Sample 1) consisted of 254 adults, of whom 124 (48.8%) were women and 130 (51.2%) were men. This sample was representative for the adult Flemish population with respect to age, gender, and level of education. Information about the distribution of gender, age and educational level of the adult Flemish population was used to determine which participants should be invited. Adults aged between 19 and 64 years were included in the study. Mean age was 39.37 years (*SD* = 11.87) and was not significantly different between women and men (*F*(1,252) = .046, *p* = .83, η^2^ = .00). With respect to civil status, 70.4% was married or living together with a romantic partner, 15.7% of participants were unmarried and lived with their parents, 7.5% was unmarried and lived alone, and 5.1% was divorced. No significant gender differences were found concerning civil status (Χ*^2^*(5) = 1.508, *p* = .91). With respect to educational level, 52.2% completed their secondary education, 45.3% completed higher education, and 2.4% completed primary education. No significant gender differences were found concerning educational level (Χ*^2^*(3) = .531, *p* = .92).

The clinical sample (Sample 2) included 60 female patients diagnosed with different types of ED. This sample has been used previously to investigate the relationship among PB, compulsive internet use, and temperament (Claes et al., 2012). A standardized clinical interview and the Eating Disorder Inventory-2 (EDI-2; Garner, 1991; Dutch version: van Strien & Ouwens, 2003) were used to determine the ED category according to the DSM-IV criteria (American Psychiatric Association, 1994). In this sample, 38.3% patients were diagnosed with anorexia nervosa restrictive type, 6.7% with anorexia nervosa binge-eating/purging type, 26.7% with bulimia nervosa, and 28.3% with eating disorder not otherwise specified. All participants were aged between 15 and 57 years, with a mean age of 27.82 years (*SD* = 9.76). Concerning age, patients with anorexia nervosa restrictive type (*M* = 23.33, *SD* = 6.87) were significantly younger than patients diagnosed with eating disorder not otherwise specified (*M* = 34, *SD* = 11.68, *F*(3,52) = 4.06, *p* < .01). The other groups did not differ significantly from each other.

When comparing Sample 1 with Sample 2, a significant difference in mean age became apparent. Although both samples covered a similar age range, participants in Sample 1 were on average significantly older compared to participants in Sample 2 (*F*(1,308) = 46.05, *p* < .001)). Moreover, Sample 1 contained both males and females, whereas Sample 2 only included females.

Sample 1 was collected by master theses students, who invited individuals to participate based on gender, age, and educational level, to obtain a representative sample for the Flemish population. Sample 2 was collected via ED patients’ individual therapists. These therapists provided patients with informed consents and questionnaires and collected them afterwards, without having knowledge of the responses of the participants. The participants themselves and the parents from minors provided written informed consent and participants completed the different questionnaires individually. Both data-collections were approved by the ethics committee of the Faculty of Psychology and Educational Sciences (SMEC) at University of Leuven.

### Instruments

We assessed the lifetime presence of NSSI by means of the NSSI subscale of the Self-Harm Inventory (SHI; Sansone et al., 1998), which determines the engagement in five different types of lifetime NSSI acts (cutting, burning, hitting, scratching and head-banging) by means of yes/no questions. The Kuder-Richardson-20 (KR-20) reliability coefficient was .43 for Sample 1 and .66 for Sample 2.

The Compulsive Buying Scale (CBS; Faber & O’Guinn, 1992) was used to measure PB. This questionnaire contains seven items, investigating behaviours and feelings related to PB. For the first item (‘If I have any money left at the end of the pay period, I just have to spend it’), participants were asked to answer using a 5-point Likert scale ranging from 1 (strongly agree) to 5 (strongly disagree). The following six items (e.g. ‘I bought myself something in order to make myself feel better’) were answered using a 5-point Likert scale ranging from 1 (very often) to 5 (never). Cronbach’s alphas were .69 for Sample 1 and .72 for Sample 2. To determine the cut-off score for PB, the authors of the CBS developed a scoring system based on a regression equation with item weighting (Faber & O’Guinn, 1992). According to a German population-based survey, a cut-off score equal to -1.09 or lower indicates the presence of PB (Mueller et al., 2010). This cut-off score of -1.09 could correctly classify 91.1% of individuals with clinical PB (Mueller et al., 2010). For the two samples included in this paper, the CBS was translated into Dutch and afterwards back-translated into English by professional translators. The original CBS scores were reversed, so that higher CBS scores pointed to higher levels of PB. In the present study, we used both the dimensional PB scores and the cut-off PB scores.

Reactivity was measured by means of the Behaviour Inhibition System (BIS) and Behaviour Activation System (BAS) Scales (BISBAS scales; Carver & White, 1994, Dutch version; Beck et al., 2009). The BISBAS scales consists of 20 items, answered by using a 4-point Likert scale ranging from 1 (I strongly agree) to 4 (I strongly disagree). The BIS scale assesses punishment sensitivity and tendency to avoid (*n* = 7; e.g. ‘Criticism or scolding hurts me quite a bit’). Cronbach’s alpha was .84 for Sample 1 and .80 for Sample 2. The BAS scale assesses reward sensitivity and tendency to approach (*n* = 13; e.g. ‘When I want something, I usually go all-out to get it’). Cronbach’s alpha was .79 for the Sample 1 and .78 for the Sample 2. Effortful Control was measured with the Effortful Control Scale from the Adult Temperament Questionnaire-Short Form (ATQ-SF-EC; Evans & Rothbart, 2007; Dutch version: Hartman & Rothbart, 2001). This scale contains 19 items (e.g. ‘I hardly ever finish things on time’). Each item is scored on a 7-point Likert scale ranging from 1 (not at all applicable) to 7 (completely applicable). Cronbach’s alpha was .76 for Sample 1 and .83 for Sample 2.

### Analyses

All analyses were performed by means of SPSS 26. To calculate the lifetime prevalence of NSSI in both samples, we calculated how many participants engaged in NSSI at least once in their life. The prevalence rates of PB were calculated based on the number of participants who scored above the cut-off score of the CBS. Spearman correlation coefficients were used to examine associations between NSSI (0/1) and dimensional PB scores/temperament, whereas Pearson correlation coefficients were used to examine associations between dimensional PB scores and temperament. Finally, we used hierarchical logistic and linear regression analysis to predict NSSI and PB, respectively, on the basis of reactivity and effortful control and their interactions, while controlling for age. Before performing regression analyses, we standardized all continuous predictors. To be able to compare female community adults, male community adults, and female patients, analyses were performed separately for these three groups across Samples 1–2. Alpha level was set at .05 for all analyses.

## Results

### NSSI

The reported lifetime prevalence of NSSI was 13.8% in the total community sample and 36.7% in the clinical sample. In the community sample, no significant gender differences were found concerning lifetime NSSI (*Χ^2^* (1) = .111, *p* = .739); 14.5% of the community women reported lifetime NSSI engagement, compared to 13.1% of the community men. However, the reported lifetime prevalence of NSSI was significantly higher in patients with ED compared to both male community adults (*Χ^2^* (1) = 14.00, *p* < .001) and female community adults (*Χ^2^* (1) = 11.66, *p* < .001). Further, participants reporting lifetime NSSI were significantly younger than participants without lifetime NSSI (*F*(1,308) = 37.29, *p* < .001). Table 1 shows the occurrence of different NSSI methods for the different groups. In the community sample, 8.7% reported the use of one NSSI method, whereas 4.7% used two different NSSI methods and 0.4% used three NSSI methods. In the clinical sample, 11.7% used one method of NSSI, whereas 15%, 6.7%, and 3.3% of patients with ED reported the use of respectively two, three, or four NSSI methods.

**Table 1:** Number of participants who have engaged in different methods of NSSI.

NSSI method	Total community sample		Female community sample		Male community sample		Female patient sample	

	*N* = 254		*n* = 124		*n* = 130		*N* = 60	

Cutting	3.1%	8	4.8%	6	1.5%	2	13.3%	8
Burning	0.0%	0	0.0%	0	0.0%	0	1.7%	1
Hitting	3.9%	10	4.0%	5	3.8%	5	23.3%	14
Head banging	9.8%	25	8.1%	10	11.5%	15	18.3%	11
Scratching	2.4%	6	4.0%	5	0.8%	1	18.3%	11

*Note*: Within group percentages are given. NSSI, non-suicidal self-injury.

### PB

In the community sample, 2.4% scored above the cut-off score for PB. However, community women (4.8%) engaged significantly more in PB compared to community men (1.5%) (*Χ^2^* (1) = 6.34, *p* < .05). In addition, 10% of female patients with ED scored above the cut-off score for PB. The patients with ED scored significantly more above the cut-off score for PB compared to male community adults (*Χ^2^* (1) = 13.22, *p* < .001), but not compared to female community adults (*Χ^2^* (1) = 1.77, *p* = .18). Also, participants scoring above the cut-off for PB were significantly younger than those scoring above the cut-off for PB (*F*(1,249) = 4.85, *p* < .05).

### Associations NSSI and PB

In Table 2 the correlations among the study variables are displayed. With respect to the association between the presence/absence of NSSI and the dimensional measured PB score, only in the female community sample the presence of NSSI was significantly positively related to the level of PB, indicating that the presence of NSSI was related to higher scores on PB in the female community sample. NSSI and PB were neither significantly related in the male community sample nor in the clinical sample.

**Table 2:** Correlations between NSSI, PB, temperamental characteristics, and age.

	Female community sample		Male community sample		Female patient sample	

	NSSI	PB	NSSI	PB	NSSI	PB

NSSI	1.00	0.33***	1.00	–0.08	1.00	–0.11
PB	0.33***	1.00	–0.08	1.00	–0.11	1.00
BIS	0.17	0.18*	0.09	0.08	0.22	–0.04
BAS	0.04	0.28**	0.00	0.11	–0.19	0.17
EC	–0.20*	–0.41***	–0.27**	–0.28**	0.09	–0.43***
Age	–0.33***	–0.15	–0.31***	–0.13	0.00	–0.13

*Note*: Associations between NSSI (0/1) and dimensional PB scores/temperament were calculated by means of Spearman correlation coefficients, whereas associations between the dimensional PB scores and temperament were calculated by means of Pearson correlation coefficients. NSSI, non-suicidal self-injury; PB, pathological buying; BIS, behavioural inhibition system; BAS, behavioural activation system; EC, effortful control. *p < .05. **p < .01 ***p < .001.

### Associations between NSSI, PB and temperament

NSSI was negatively related to age and effortful control in community women and men. No significant associations were found between NSSI and BIS/BAS reactivity or effortful control in the clinical sample. Further, PB was negatively related to effortful control in the three groups. PB was also positively related to BIS and BAS in community women. No significant relationship was found between PB and age.

### Prediction of NSSI

**Figure 1:**
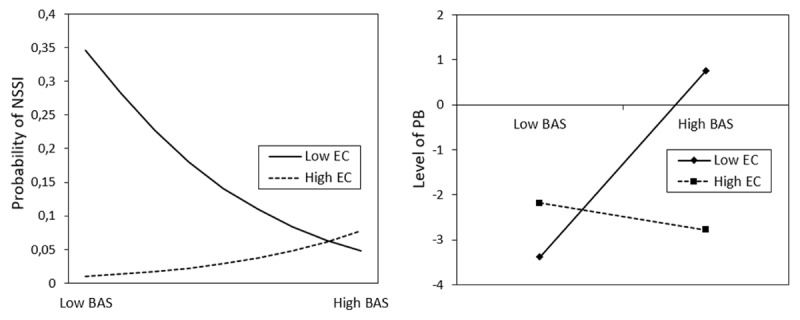
Interaction between BAS and effortful control in the prediction of NSSI for community men and in the prediction of PB for community women. NSSI; non-suicidal self-injury; PB, pathological buying; BAS, behavioural activation system; EC, effortful control.

Table 3 displays the results of the logistic regression analyses with BIS/BAS reactivity and effortful control and their interactions as predictors for NSSI, controlling for age. In community women, NSSI was negatively predicted by age. In community men, NSSI was negatively predicted by age and positively predicted by the interaction between BAS and effortful control. This interaction indicated that community men with low BAS and low effortful control have the highest probability to engage in NSSI (see Figure 1). Finally, in the clinical sample, NSSI was negatively predicted by BAS: a lower level of BAS was related to more engagement in NSSI.

**Table 3:** Prediction of NSSI based on reactivity and effortful control and their interactions, controlled for age.

Step	Variables in regression	Female community sample		Male community sample		Female patient sample	

		*B*	Nagelkerke *R^2^*	*B*	Nagelkerke *R^2^*	*B*	Nagelkerke *R^2^*

Step 1	Age	–0.96**	0.16	–1.21**	0.21	–0.15	0.01
Step 2	Age	–0.80*		–1.19**		–0.32	
	BIS	0.54		0.40		0.50	
	BAS	–0.36		–0.39		–0.63	
	EC	–0.47	0.22	–0.51	0.29	–0.04	0.14
Step 3	Age	–0.88*		–1.27**		–0.47	
	BIS	0.51		0.28		0.58	
	BAS	–0.40		–0.06		–0.81*	
	EC	–0.58		–0.86		–0.17	
	BIS*EC	0.03		–0.11		0.493	
	BAS*EC	–0.52	0.27	1.11*	0.35	–0.632	0.28

*Note*: NSSI, non-suicidal self-injury; BIS, behavioural inhibition system; BAS, behavioural activation system; EC, effortful control. * *p* < .05. **p < .01.

### Prediction of PB

Table 4 presents the results of the linear regression analyses with BIS/BAS reactivity and effortful control and their interactions as predictors for PB, controlling for age. Regression analyses indicated that PB was predicted by low effortful control in all three groups. In community women, PB was positively predicted by BAS, and negatively predicted by the interaction between BAS and effortful control. This interaction indicated that community women with high BAS and low effortful control displayed the highest levels of PB (see Figure 1).

**Table 4:** Prediction of PB based on reactivity and effortful control and their interactions, controlled for age.

Step	Variables in regression	Female community sample		Male community sample		Female patient sample	

		*B*	*R^2^*	*B*	*R^2^*	*β*	*R^2^*

Step 1	Age	–0.14	0.02	–0.09	0.01	–0.12	0.02
Step 2	Age	0.05		0.04		–0.01	
	BIS	0.13		–0.01		–0.03	
	BAS	0.18*		0.05		0.12	
	EC	–0.37**	0.23**	–0.27*	0.07	–0.44**	0.23**
Step 3	Age	0.05		0.06		0.01	
	BIS	0.12		–0.02		–0.04	
	BAS	0.20*		0.04		0.12	
	EC	–0.36**		–0.38*		–0.42*	
	BIS*EC	–0.00		–0.14		–0.16	
	BAS*EC	–0.24**	0.28*	0.11	0.10	0.11	0.26

*Note*: PB, pathological buying; BIS, behavioural inhibition system; BAS, behavioural activation system; EC, effortful control. * *p* < .05. **p < .01.

## Discussion

In this study, the (co-)occurrence of NSSI and PB was investigated in a sample of female patients with ED and in a sample of community adults. The lifetime prevalence of NSSI was significantly higher in the ED patient sample (36.7%) compared to the community sample (13.8%). This result is consistent with previous research findings indicating a lifetime prevalence of NSSI between 25% and 55% in patients with ED (Pérez et al., 2018) and between 2% and 22% in community adults (Swannell et al., 2014). Further, the present study demonstrated that more patients with ED scored above the cut-off for PB (10%) compared to community men (1.5%), but not compared to community women (4.8%). This prevalence rate of PB in patients with ED is in line with the prevalence rate around 12% found in other ED patient samples (Fernandez-Aranda et al., 2008), whereas the prevalence rate for community adults (2.4%) is somewhat lower than the prevalence rate around 5% reported in other community samples (Maraz et al., 2016). A possible explanation for this might be that previous studies included more women, and, in accordance with the present results, these studies demonstrated that women are more vulnerable for PB engagement than men (Maraz et al., 2016).

Additionally, the present study investigated the association between NSSI and PB in different groups. Although both behaviours occurred more frequently in the ED patient sample, no significant association between NSSI and PB was found in this group, which is in contrast to our expectations. It seems possible that PB and NSSI are driven by different underlying mechanisms in patients with ED. Also, this outcome may be influenced by the high levels of patients diagnosed with anorexia nervosa restrictive type (38.3%) in our clinical sample. Given the particularly high occurrence of PB and NSSI in patients with bulimia nervosa or eating disorder not otherwise specified (Fernandez-Aranda et al., 2008; Pérez et al., 2018), the outcome might be different in these subsamples of ED. Future research including larger samples of patients with ED, with a balanced number of different subtypes of ED, is required. Apart from that, we found a statistically significant positive relationship between NSSI and PB in the female community sample. This result indicates that community women who engage in one or more NSSI behaviours also report higher levels of PB, suggesting the co-occurrence of these pathological behaviours in community women. An implication of this outcome is the possibility that community women share vulnerability factors for NSSI and PB, such as emotion regulation problems or similar underlying temperamental characteristics.

To gain insight in the temperamental vulnerabilities underlying NSSI and PB in different groups, we investigated whether NSSI and PB are characterized by similar/different temperamental characteristics in female patients with ED and in male and female community adults. Engagement in NSSI was related to low levels of self-regulation in the male and female community sample. This result is consistent with our expectations based on other studies in community individuals (Baetens et al., 2011; Gandhi et al., 2016). Interestingly, NSSI was positively predicted by the interaction of BAS reactivity and effortful control in the male community sample, indicating that the association between NSSI and behavioural activation is moderated by effortful control in this group. More specifically, community men with low levels of BAS reactivity and low levels of effortful control are the most likely to engage in NSSI. Similarly, we found that NSSI was negatively predicted by BAS reactivity in the ED patient sample, which indicates that patients with ED with low BAS reactivity (i.e., anhedonia), are more vulnerable to engage in NSSI. Although previous studies found NSSI and BAS reactivity to be unrelated (Gandhi et al., 2016), the negative association that was found in the present study may be partly explained by the association between NSSI and depressive symptoms that has been demonstrated before (Wilkinson & Goodyer, 2011). Depression is characterized by high BIS reactivity, low BAS reactivity and low effortful control (Bijttebier et al., 2009). Importantly, low BAS reactivity in depression is associated with feelings of anhedonia and inactivity (Kasch et al., 2002). It may be that individuals with low BAS reactivity engage in NSSI to stimulate themselves to feel something (even if this feeling is pain), which is in accordance with the finding of Nock and Prinstein (2005) that depressive individuals engage in NSSI to compensate for feelings of anhedonia, emptiness and detachment. However, inconsistent with our predictions, we found no association between NSSI and BIS reactivity or effortful control in the ED patient sample. A possible explanation for this result might be that NSSI in the ED patient sample is different in nature than NSSI in the community sample. Apart from a higher prevalence of lifetime NSSI in patients with ED, the extent of severe and recent NSSI is probably also higher in patients with ED compared to community adults. A recent study of Buelens et al. (2020), identified different temperamental characteristics in patients with ED reporting only lifetime NSSI versus patients with ED also reporting recent NSSI. Consequently, including measurements of recent NSSI in future research is suggested to improve understanding about NSSI and its underlying mechanisms.

With respect to the association between PB and temperamental characteristics, engagement in PB was predicted by low levels of self-regulation in all three groups. This result supports previous studies that have found that self-regulatory resources are related to PB behaviours. Individuals with self-regulatory difficulties were more likely to spend more money than intended and to feel a stronger urge to buy, compared to individuals without self-regulatory difficulties (Vohs & Faber, 2007). Although it has been suggested that PB and BIS reactivity are positively associated in community men (Mueller et al., 2011), the present study did not confirm this finding. Remarkably, PB was found to be related to high levels of reactivity (BIS/BAS) and low levels of effortful control in community women. Finally, the present study confirms that PB is positively predicted by the interaction of behavioural activation and effortful control in community women. This means that effortful control has a moderating role in the regulation of BAS reactivity in PB. Community women with high levels of BAS reactivity and low levels of effortful control, are more vulnerable for PB. These findings indicate that especially community women with low effortful control are driven by the tendency to experience positive feelings while buying, which is in line with previous findings regarding underlying motivational processes of buying in community individuals (Dittmar et al., 1996).

In sum, whereas NSSI and PB had different associations with BIS/BAS reactivity, both behaviours were negatively associated with effortful control in the different groups. Significant associations were found between both PB and NSSI and lack of effortful control, which could be a possible explanation for the co-morbidity of these behaviours with other psychiatric disorders characterized by low effortful control (such as EDs and borderline personality disorder). Because both PB and NSSI are predicted by problems with effortful control in different groups, the treatment of these pathological behaviours should include training in this top-down control. An improvement in effortful control could ideally address different pathological behaviours at the same time. Previous studies already demonstrated a significant improvement in effortful control after specific treatment interventions (Faber & Vohs, 2004; Prins et al., 2013). In addition to some more classical self-regulation training modules (Baumeister & Heatherton, 1996), more modern tools such as the videogame ‘Playmancer’ developed by Jiménez-Murcia et al. (2009), are promising for the improvement of self-regulation capacities.

This study was, to the best of our knowledge, one of the first studies investigating the association between NSSI and PB and their underlying temperamental characteristics. Furthermore, it was the first study investigating this research topic in both a community sample and a clinical ED sample. However, some limitations should be recognized when interpreting the results. A first limitation is the method we used to assess the different study variables, which is limited to self-report instruments. Future research should benefit from the use of diverse measurements, such as standardized clinical interviews to assess pathological behaviours, or behavioural and performance-based tasks to assess temperamental characteristics. Additionally, assessing these pathological behaviours in several clinical groups would allow future research to investigate NSSI and PB while taking relevant comorbid psychopathology into account (e.g., borderline personality disorder, EDs, or substance use disorder). Describing comorbid psychopathology could elaborate the study results by further characterising those engaging in NSSI and/or PB.

A second limitation are the relative small sample sizes of both samples. The replication of our results in larger samples of patients with ED, NSSI or the primary diagnosis PB, and in larger community samples will be needed. Moreover, our descriptive results comparing the clinical to the community sample should be interpreted tentatively. More specifically, although our results do provide a relevant indication of clinical and community NSSI/PB prevalence rates, the descriptive results were limited by gender and age differences between the samples. These age and gender differences warrant careful comparison of prevalence rates between both samples, as potential differences could be influenced by or due to the younger, female participants in Sample 2. Previous research has shown that these differences are relevant for NSSI and PB, as younger and female individuals are more likely to exhibit both NSSI and PB (Maraz et al., 2016; Plener et al., 2015). Hence, future studies should use more balanced clinical and community samples in terms of age and gender to allow for more authoritative conclusions. Finally, the cross-sectional nature of this study prevented us to investigate the direction of associations between the different study variables. Future longitudinal studies should be conducted to determine the directionality of these associations.

## References

[1] American Psychiatric Association. (1994). Diagnostic and statistical manual of psychiatric disorders. Washington, D.C.: Author.

[2] Baetens, I., Claes, L., Willem, L., Muehlenkamp, J., & Bijttebier, P. (2011). The relationship between non-suicidal self-injury and temperament in male and female adolescents based on child- and parent-report. Personality and Individual Differences, 50, 527–530. DOI: 10.1016/j.paid.2010.11.015

[3] Baumeister, R. F., & Heatherton, T. F. (1996). Self-regulation failure: An overview. Psychological Inquiry, 7, 1–15. DOI: 10.1207/s15327965pli0701_1

[4] Beck, I., Smits, D. J., Claes, L., Vandereycken, W., & Bijttebier, P. (2009). Psychometric evaluation of the behavioral inhibition/behavioral activation system scales and the sensitivity to punishment and sensitivity to reward questionnaire in a sample of eating disordered patients. Personality and Individual Differences, 47, 407–412. DOI: 10.1016/j.paid.2009.04.007

[5] Bijttebier, P., Beck, I., Claes, L., & Vandereycken, W. (2009). Gray’s Reinforcement Sensitivity Theory as a framework for research on personality–psychopathology associations. Clinical Psychology Review, 29, 421–430. DOI: 10.1016/j.cpr.2009.04.002 19403216

[6] Buelens, T., Luyckx, K., Gandhi, A., Kiekens, G., & Claes, L. (2019). Non-suicidal self-injury in adolescence: Longitudinal associations with psychological distress and rumination. Journal of Abnormal Child Psychology, 47, 1569–1581. DOI: 10.1007/s10802-019-00531-8 30900112

[7] Buelens, T., Luyckx, K., Verschueren, M., Schoevaerts, K., Dierckx, E., Depestele, L., & Claes, L. (2020). Temperament and character traits of female eating disorder patients with (out) non-suicidal self-injury. Journal of Clinical Medicine, 9, 1207 DOI: 10.3390/jcm9041207

[8] Carver, C. S., & White, T. L. (1994). Behavioral inhibition, behavioral activation, and affective responses to impending reward and punishment: The BIS/BAS scales. Journal of Personality and Social Psychology, 67, 319 DOI: 10.1037/0022-3514.67.2.319

[9] Claes, L., Bijttebier, P., Mitchell, J. E., de Zwaan, M., & Mueller, A. (2011). The relationship between compulsive buying, eating disorder symptoms, and temperament in a sample of female students. Comprehensive Psychiatry, 52, 50–55. DOI: 10.1016/j.comppsych.2010.05.003 21220065

[10] Claes, L., Bijttebier, P., Van Den Eynde, F., Mitchell, J. E., Faber, R., de Zwaan, M., & Mueller, A. (2010). Emotional reactivity and self-regulation in relation to compulsive buying. Personality and Individual Differences, 49, 526–530. DOI: 10.1016/j.paid.2010.05.020

[11] Claes, L., Muller, A., Norre, J., Van Assche, L., Wonderlich, S., & Mitchell, J. E. (2012). The relationship among compulsive buying, compulsive internet use and temperament in a sample of female patients with eating disorders. European Eating Disorder Review, 20, 126–131. DOI: 10.1002/erv.1136 21710571

[12] Claes, L., Norré, J., Van Assche, L., & Bijttebier, P. (2014). Non-suicidal self-injury (functions) in eating disorders: Associations with reactive and regulative temperament. Personality and Individual Differences, 57 DOI: 10.1016/j.paid.2013.09.022

[13] Derryberry, D., & Rothbart, M. K. (1997). Reactive and effortful processes in the organization of temperament. Development and Psychopathology, 9, 633–652. DOI: 10.1017/S0954579497001375 9448999

[14] Dittmar, H., Beattie, J., & Friese, S. (1996). Objects, decision considerations and self-image in men’s and women’s impulse purchases. Acta Psychologica, 93, 187–206. DOI: 10.1016/0001-6918(96)00019-4 8826795

[15] Evans, D. E., & Rothbart, M. K. (2007). Developing a model for adult temperament. Journal of Research in Personality, 41, 868–888. DOI: 10.1016/j.jrp.2006.11.002

[16] Faber, R. J., & O’guinn, T. C. (1992). A clinical screener for compulsive buying. Journal of consumer Research, 19(3), 459–469. DOI: 10.1086/209315

[17] Faber, R. J., & Vohs, K. D. (2004). To Buy or Not to Buy? Handbook of self-regulation, 509–524.

[18] Fernandez-Aranda, F., Pinheiro, A. P., Thornton, L. M., Berrettini, W. H., Crow, S., Fichter, M. M., … Bulik, C. M. (2008). Impulse control disorders in women with eating disorders. Psychiatry Research, 157, 147–157. DOI: 10.1016/j.psychres.2007.02.011 17961717

[19] Gandhi, A., Luyckx, K., Maitra, S., Kiekens, G., & Claes, L. (2016). Reactive and regulative temperament and non-suicidal self-injury in Flemish adolescents: The intervening role of identity formation. Personality and Individual Differences, 99, 254–259. DOI: 10.1016/j.paid.2016.05.007

[20] Garner, D. M. (1991). EDI-2: Eating disorder inventory-2. Odessa: Psychological Assessment Resources.

[21] Gray, J. A. (1991). The neuropsychology of temperament In Explorations in temperament (pp. 105–128): Springer DOI: 10.1007/978-1-4899-0643-4_8

[22] Hartman, C., & Rothbart, M. (2001). Dutch version of the Adult Temperament Questionnaire-Short Form Groningen, NL: Groningen Faculteit der Medische Wetenschappen.

[23] Jenkins, A. L., Seelbach, A. C., Conner, B. T., & Alloy, L. B. (2013). The roles of behavioural activation and inhibition among young adults engaging in self-injury: The roles of BAS and BIS in NSSI. Personality and Mental Health, 7, 39–55. DOI: 10.1002/pmh.1200 24343924 PMC4066467

[24] Jiménez-Murcia, S., Fernández-Aranda, F., Kalapanidas, E., Konstantas, D., Ganchev, T., Kocsis, O., … Breiteneder, C. (2009). Playmancer project: a serious videogame as an additional therapy tool for eating and impulse control disorders. Studies in Health Technology and Informatics, 163–166. DOI: 10.3233/978-1-60750-017-9-163 19592756

[25] Jiménez-Murcia, S., Steiger, H., Isräel, M., Granero, R., Prat, R., Santamaría, J. J., … Fernández-Aranda, F. (2013). Pathological gambling in eating disorders: Prevalence and clinical implications. Comprehensive Psychiatry, 54, 1053–1060. DOI: 10.1016/j.comppsych.2013.04.014 23759149

[26] Kasch, K. L., Rottenberg, J., Arnow, B. A., & Gotlib, I. H. (2002). Behavioral activation and inhibition systems and the severity and course of depression. Journal of Abnormal Psychology, 111, 589 DOI: 10.1037//0021-843X.111.4.589 12428772

[27] Kyrios, M., McQueen, P., & Moulding, R. (2013). Experimental analysis of the relationship between depressed mood and compulsive buying. Journal of Behavior Therapy and Experimental Psychiatry, 44, 194–200. DOI: 10.1016/j.jbtep.2012.10.004 23207967

[28] Lejoyeux, M., & Weinstein, A. (2010). Compulsive buying. The American Journal of Drug and Alcohol Abuse, 36, 248–253. DOI: 10.3109/00952990.2010.493590 20560822

[29] Maraz, A., Griffiths, M. D., & Demetrovics, Z. (2016). The prevalence of compulsive buying: A meta-analysis. Addiction, 111, 408–419. DOI: 10.1111/add.13223 26517309

[30] McElroy, S. L., Keck, P. E., Pope, H. G., Smith, J. M., & Strakowski, S. M. (1994). Compulsive buying: A report of 20 cases. The Journal of clinical psychiatry. 8071278

[31] Muehlenkamp, J. J., Peat, C. M., Claes, L., & Smits, D. (2012). Self-injury and disordered eating: Expressing emotion dysregulation through the body. Suicide and Life-Threatening Behavior, 42, 416–425. DOI: 10.1111/j.1943-278X.2012.00100.x 22646483

[32] Mueller, A., Claes, L., Mitchell, J. E., Faber, R. J., Fischer, J., & de Zwaan, M. (2011). Does compulsive buying differ between male and female students? Personality and Individual Differences, 50, 1309–1312. DOI: 10.1016/j.paid.2011.02.026

[33] Mueller, A., Mitchell, J. E., Crosby, R. D., Gefeller, O., Faber, R. J., Martin, A., … de Zwaan, M. (2010). Estimated prevalence of compulsive buying in Germany and its association with sociodemographic characteristics and depressive symptoms. Psychiatry Research, 180, 137–142. DOI: 10.1016/j.psychres.2009.12.001 20494451

[34] Nock, M. K. (2009). Understanding nonsuicidal self-injury: Origins, assessment, and treatment: American Psychological Association DOI: 10.1037/11875-000

[35] Nock, M. K., & Prinstein, M. J. (2005). Contextual features and behavioral functions of self-mutilation among adolescents. Journal of Abnormal Psychology, 114, 140–146. DOI: 10.1037/0021-843X.114.1.140 15709820

[36] Pérez, S., Marco, J. H., & Cañabate, M. (2018). Non-suicidal self-injury in patients with eating disorders: Prevalence, forms, functions, and body image correlates. Comprehensive Psychiatry, 84, 32–38. DOI: 10.1016/j.comppsych.2018.04.003 29679850

[37] Plener, P. L., Schumacher, T. S., Munz, L. M., & Groschwitz, R. C. (2015). The longitudinal course of non-suicidal self-injury and deliberate self-harm: A systematic review of the literature. Borderline personality disorder and emotion dysregulation, 2, 2 DOI: 10.1186/s40479-014-0024-3 26401305 PMC4579518

[38] Prins, P. J., Brink, E. T., Dovis, S., Ponsioen, A., Geurts, H. M., De Vries, M., & Van Der Oord, S. (2013). “Braingame Brian”: Toward an executive function training program with game elements for children with ADHD and cognitive control problems. GAMES FOR HEALTH: Research, Development, and Clinical Applications, 2, 44–49. DOI: 10.1089/g4h.2013.0004 26196554

[39] Ribeiro, J. D., Franklin, J. C., Fox, K. R., Bentley, K. H., Kleiman, E. M., Chang, B. P., & Nock, M. K. (2016). Self-injurious thoughts and behaviors as risk factors for future suicide ideation, attempts, and death: A meta-analysis of longitudinal studies. Psychological Medicine, 46, 225–236. DOI: 10.1017/S0033291715001804 26370729 PMC4774896

[40] Rothbart, M. K., Ahadi, S. A., & Evans, D. E. (2000). Temperament and personality: Origins and outcomes. Journal of Personality and Social Psychology, 78, 122 DOI: 10.1037//0022-3514.78.1.122 10653510

[41] Sansone, R. A., Wiederman, M. W., & Sansone, L. A. (1998). The self-harm inventory (SHI): Development of a scale for identifying self-destructive behaviors and borderline personality disorder. Journal of Clinical Psychology, 54, 973–983. DOI: 10.1002/(SICI)1097-4679(199811)54:7<973::AID-JCLP11>3.0.CO;2-H 9811134

[42] Swannell, S. V., Martin, G. E., Page, A., Hasking, P., & St John, N. J. (2014). Prevalence of nonsuicidal self-injury in nonclinical samples: Systematic review, meta-analysis and meta-regression. Suicide and Life-Threatening Behavior, 44, 273–303. DOI: 10.1111/sltb.12070 24422986

[43] van Strien, T., & Ouwens, M. (2003). Validation of the Dutch EDI-2 in one clinical and two nonclinical populations. European Journal of Psychological Assessment, 19(1), 66 DOI: 10.1027//1015-5759.19.1.66

[44] Vohs, K. D., & Faber, R. J. (2007). Spent resources: Self-regulatory resource availability affects impulse buying. Journal of Consumer Research, 33, 537 DOI: 10.1086/510228

[45] Weinstein, A., Maraz, A., Griffiths, M. D., Lejoyeux, M., & Demetrovics, Z. (2016). Compulsive buying: Features and characteristics of addiction In Neuropathology of drug addictions and substance misuse (pp. 993–1007): Elsevier DOI: 10.1016/B978-0-12-800634-4.00098-6

[46] Welch, S. S., Linehan, M. M., Sylvers, P., Chittams, J., & Rizvi, S. L. (2008). Emotional responses to self-injury imagery among adults with borderline personality disorder. Journal of Consulting and Clinical Psychology, 76, 45 DOI: 10.1037/0022-006X.76.1.45 18229982

[47] Wilkinson, P., & Goodyer, I. (2011). Non-suicidal self-injury. European Child and Adolescent Psychiatry, 20, 103–108. DOI: 10.1007/s00787-010-0156-y 21222215

